# Reducing enteric methane emission in dairy goats: impact of dietary inclusions of quebracho tannin extract on ruminal microbiota

**DOI:** 10.3389/fmicb.2025.1595924

**Published:** 2025-07-07

**Authors:** Paola Cremonesi, Marco Severgnini, Marco Battelli, Valentina Monistero, Martina Penati, Alessia Libera Gazzonis, Bianca Castiglioni, Luca Rapetti, Maria Teresa Manfredi, Maria Filippa Addis

**Affiliations:** ^1^Institute of Agricultural Biology and Biotechnology – National Research Council (IBBA-CNR), Lodi, Italy; ^2^Institute of Biomedical Technologies – National Research Council (ITB-CNR), Segrate (MI), Italy; ^3^National Biodiversity Future Center S.c.a.r.l., Palermo, Italy; ^4^Department of Agricultural and Environmental Sciences – Production, Landscape, Agroenergy, University of Milan, Milan, Italy; ^5^Department of Veterinary Medicine and Animal Sciences, University of Milan, Lodi, Italy; ^6^Research Laboratory of Animal Parasitic Diseases and Zoonoses (Parvetlab), University of Milan, Lodi, Italy; ^7^Laboratory of Animal Infectious Diseases (MiLab), University of Milan, Lodi, Italy

**Keywords:** methanogenesis, rumen fermentation, condensed tannins, environmental sustainability, microbiota modulation

## Abstract

**Introduction:**

Condensed tannins (CT) influence ruminal microbiota, feed digestibility, and methane emissions, yet their effects in goats are poorly understood.

**Methods:**

This study evaluated the impact of dietary quebracho CT extract at 0%, 2%, 4%, or 6% of dry matter on the composition of the dairy goat ruminal microbiota with a two-times repeated 4 × 4 Latin square design. Bacterial, archaeal, fungal, and protozoan communities were analyzed at the end of each feeding period for relative abundance changes, and their relationship to methane production, nutrient digestibility and feed efficiency were also assessed.

**Results:**

Increasing CT levels reduced alpha- and beta-diversity, with the 6% CT diet showing the most pronounced decline. CT inclusion induced phylum-level shifts in fiber-degrading microbes, including inversion of the Firmicutes to Bacteroidota ratio. Prevotellaceae and Succiniclasticum, tolerant to CT, increased significantly (*P* < 0.05), in line with higher propionate and lower methane production. The proteolytic bacteria Anaerolineaceae and Synergistaceae decreased (*P* < 0.05), consistently with the reduced isobutyrate and isovalerate ruminal concentration and with the reduced urinary nitrogen excretion. *Methanobrevibacter*, a key methane producer, was reduced by dietary CT (*P* < 0.05). The overall fungal biodiversity was also significantly changed (*P* < 0.05); the fiber-degrading *Liebetanzomyces* decreased, while the tannin-degrading *Aspergillus* increased (*P* < 0.05). Concerning protozoa, *Diplodinium* was reduced (*P* < 0.05) and *Polyplastron* and *Isotrichia* were increased (*P* < 0.05) by dietary CT.

**Discussion:**

These and other microbial abundance changes correlated with reduced methane emission, altered fiber and protein digestibility, and modified volatile fatty acid (VFA) profiles. This study shows that decreased nutrient degradability in the rumen due to higher dietary CT alters the goat rumen microbiota and clarifies microbial taxa changes in relation to the zootechnical outcomes, including reduced methane production.

## 1 Introduction

The rumen microbiota structure, both in terms of taxonomic groups and community states, clearly influences fermentation efficiency and nutrients digestibility, as well as methane production (Morgavi et al., 2010; Moraïs and Mizrahi, 2019b). A proposed mitigation strategy is dietary supplementation with tannins (Aboagye and Beauchemin, 2019). These secondary plant metabolites belong to the class of polyphenols and are generally classified into hydrolyzable tannins (HT) and condensed tannins (CT) (Besharati et al., 2022). Research has focused more on CT because of the toxicity associated with HT degradation products (Naumann et al., 2017). One of their main properties is the ability to form complexes with proteins and protect them from ruminal degradation (Hagerman et al., 1992). Since urinary nitrogen is significantly more labile and prone to rapid leaching and volatilization losses compared to fecal nitrogen (Hristov et al., 2019), the affinity between CT and dietary proteins can play a positive role for the environment due to the shift from urinary to fecal nitrogen excretion (Mueller-Harvey et al., 2019; Hristov et al., 2022; Battelli et al., 2024). Tannins also bind carbohydrates, particularly hemicelluloses and cellulose, as well as starch and pectin (Besharati et al., 2022). Tannins fed to ruminants can have both positive and negative effects. By acting as modulators of the rumen ecosystem and animal physiology, tannins can improve production and reproductive performance, animal health, efficiency of nitrogen utilization, and quality of derived livestock products, reducing at the same time the risk of meteorism, gastrointestinal parasite load, and environmental impact in terms of both methanogenesis and nitrogen excretion (Mueller-Harvey et al., 2019). Also, these compounds can have an anti-microbial activity when consumed by the animals and limit the growth of different microbial groups in the rumen. However, by decreasing diet digestibility, high tannin concentrations may reduce voluntary dry matter intake (DMI). Nevertheless, low to moderate concentrations may improve the digestive utilization of feed (Frutos et al., 2004; Silva de Sant'ana et al., 2022). This result may be directed by substrate modulation with the enrichment in microbial communities that carry out preferred metabolic pathways leading to the production of more desirable metabolites through nutritional intervention (Moraïs and Mizrahi, 2019b). Therefore, a better understanding of the impact of dietary integration with tannins on the gut microbiota in terms of diversity, taxonomic groups, and functional states is needed (Moraïs and Mizrahi, 2019b; Mizrahi et al., 2021).

Most studies on methane mitigation by tannins have been carried out in cows. The microbial community structure differs between goats and cows; furthermore, goats are able to detoxify tannins, polyphenols, and other secondary metabolites (Giger-Reverdin et al., 2020). Among other ruminants, goats are especially able to feed on a broad range of plant species including forages of lower quality, as these animals appear particularly resistant to ingestion of large amounts of anti-nutritional compounds and even of toxic metabolites (Silanikove et al., 1996).

Recent metagenomic studies have cataloged over 5,000 microbial genomes in the Caprinae subfamily, revealing taxonomic diversity dominated by Bacteroidota and Firmicutes (Zhang et al., 2022). Dietary interventions, such as tannin supplementation, modulate this microbiota, influencing fermentation and methanogenesis (Silva de Sant'ana et al., 2022; Zhu et al., 2025). Understanding these dynamics is essential for optimizing dairy goat production and reducing environmental impacts (Belanche et al., 2023). In a recent dedicated field study, Battelli et al. (2024) assessed the impact of increasing dietary levels of quebracho (*Schinopsis balansae*) CT extract on DMI, total tract digestibility, rumen fermentation, milk production, gas exchange, plasma metabolites, enzymatic activities, and energy and nitrogen balances of dairy goats. The authors reported a reduction in nutrient digestibility and feed use efficiency caused by the inclusion of quebracho CT. In addition, they observed a decrease in methane yield (g CH_4_/kg DMI) but no effect of CT on methane intensity when expressed per kilogram of fat- and protein-corrected milk. These findings led the authors to hypothesize that the observed reduction in methanogenesis could be related to changes in diet digestibility, with potential consequences on the rumen microbiota composition.

This study aimed to characterize the shifts in the ruminal prokaryotic and eukaryotic microbiota of dairy goats fed increasing levels of quebracho CT extract and to correlate these changes with the zootechnical parameters evaluated during the feeding trial.

## 2 Materials and methods

### 2.1 Experimental design and diet formulations

The feeding trial was carried out at “Cascina Baciocca” of the Milan University Farms Functional Centre located in Cornaredo (Milan, Italy), as detailed previously (Battelli et al., 2024). Briefly, eight multiparous lactating alpine goats having comparable parity, days in milk (DIM), and body weight (BW), were divided into two groups of four animals each. A parasitological analysis was performed before the study. A fecal sample (5 g) from each goat was collected from the rectal ampulla and kept at refrigerated temperature during transport to the laboratory. Within 48 h from the collection, a quality-quantitative copromicroscopic analysis by FLOTAC dual technique was implemented, using as flotation solutions saturated sodium chloride (NaCl, specific gravity 1.200) for the detection of nematode and cestode eggs and *Eimeria* oocysts, and zinc sulfate (ZnSO_4_, specific gravity 1.350) for the isolation of trematode eggs and lungworm first stage larvae; the number of eggs, oocysts (OPG), and larvae per gram of feces was calculated (Cringoli et al., 2010). Since helminthic elements were not detected in the analyzed samples, with the only presence of a very low number of *Eimeria* spp. oocysts (mean OPG ± standard deviation: 123.8 ± 191.11), the selected goats were enrolled for the study and no anticoccidial treatment was given.

The mean ± SD DIM was 42 ± 5, the BW was 67.1 ± 4.8 kg, and the milk yield 3.42 ± 0.55 kg/d at the beginning of the study. The experimental diets were administered twice daily, and the goats were fed *ad libitum* with free access to drinking water. The experimental diets contained the same basal ration (C) with the addition of three different concentrations of 2%, 4%, and 6% on DM of the diet (Q2, Q4, and Q6, respectively) of a commercial purified quebracho (*Schinopsis balansae*) CT extract (Silvafeed Q powder, by Silvateam, San Michele Mondovì, Cuneo, Italy) with a total CT concentration of 68.3%, corresponding to 74.1% on DM. A detailed description of the metabolomic profile of the quebracho CT extract has been provided previously (Gazzonis et al., 2023). A two-times repeated Latin square design balanced for the carry-over effect was implemented for diet administration (2 squares × 4 goats × 4 treatments). The first square was complete, while the second one was an uncompleted square because one of the four goats was removed from the trial due to a bad adaptation to the metabolic cage and two other goats were removed in two different periods, one due to mastitis in the last period, and the other one for problems with the catheter. Each experimental period was 28 days, with 24 days of adaptation followed by 4 days of data collection. During these 4 days of data collection, the goats were confined into the individual metabolic cage put inside the individual open-circuit respiration chamber. Inside the individual open-circuit respiration chamber, four 24 h cycles of respiratory exchange were measured for each goat in each period.

### 2.2 Ruminal sample collection

The ruminal samples were collected on the last day of each experimental period. Ruminal fluid was collected 5 h after the morning feeding using an esophageal semi-rigid probe with an apical ogive with small holes. To avoid saliva contamination, the first collected rumen sample was discarded. The pH was measured immediately after sampling and an aliquot was stored at −20°C until transported to the laboratory in refrigerated conditions. Once at the laboratory, the samples were stored at −80°C until lyophilization. Due to issues with animal compliance with the feeding trial, we could not collect two C, one Q4, and one Q6 rumen samples.

### 2.3 DNA extraction and purification

DNA extraction was carried out on 0.25 g of lyophilized rumen fluid following the protocol described previously (Yu and Morrison, 2004). To ensure the reliability of the extraction process, both negative and positive extraction controls were included. Sterile water was used as a negative control to monitor for potential contamination, while a standardized microbial community sample (ZymoBiomics, Zymo Research, EuroClone S.p.A., Milan, Italy) was used as a positive control to confirm the efficiency and consistency of the DNA extraction protocol. The quality and quantity of DNA samples were assessed using a NanoDrop ND-1000 spectrophotometer (NanoDrop Technologies, Wilmington, DE, USA). The isolated DNA was stored at −20°C until use.

### 2.4 16S rRNA gene library preparation and sequencing

Bacterial/Archaeal DNA was amplified according the “16S Metagenomic Sequencing Library Preparation” protocol (https://support.illumina.com/documents/documentation/chemistry_documentation/16s/16s-metagenomic-library-prep-guide-15044223-b.pdf) by Illumina (San Diego, CA, USA) using the primers described previously (Klindworth et al., 2013) which target the V3–V4 hypervariable regions of the 16S rRNA gene, while Protozoa DNA was amplified using the primers targeting 18S rRNA (Saminathan et al., 2017). For the fungal community, the ITS1-ITS2 region was used as a target (primer forward = CTTGGTCATTTAGAGGAAGTAA; primer reverse = GCTGCGTTCTTCATCGATGC) (Walters et al., 2016). The PCR amplifications, for all three targets, were performed in 25 μL volume per sample. A total of 12.5 μL of KAPA HIFI Master Mix 2 × (Kapa Biosystems, Inc., Wilmington, MA, USA) and 0.2 μL of each primer (100 μM) were added to 2 μL of genomic DNA (5 ng/μL). Blank controls (i.e., no DNA template added to the reaction) were also performed. No negative controls for DNA extraction were run. A first amplification step was performed in an Applied Biosystem 2,700 thermal cycler (ThermoFisher Scientific, Waltham, MA, USA); the samples were denatured at 95°C for 5 min, followed by 25 cycles with a denaturing step at 95°C for 1 min, annealing at 56°C for 1 min and extension at 72°C for 1 min, with a final extension at 72°C for 7 min. Amplicons were cleaned with Agencourt AMPure XP (Beckman, Coulter Brea, CA, USA) and libraries were prepared following the Metagenomic Sequencing Library Preparation Protocol (Illumina). The libraries obtained were quantified by Real Time PCR with KAPA Library Quantification Kits (Kapa Biosystems, Inc., MA, USA), pooled in equimolar proportion and sequenced in MiSeq (Illumina) with 2 × 250-base paired-end reads each.

### 2.5 Bioinformatics and biostatistics

Raw sequencing reads were processed through a bioinformatics filtering pipeline comprising the merging of the two paired reads coming from the same fragment in one single sequence by PandaSeq (Masella et al., 2012) and the trimming of low-quality bases (Phred quality score <3) from the 3′-end of each read; if resulting fragments had a length <75% of the initial length, they were discarded. Fragments were retained if they had a length over 250 nucleotides (nt) for the prokaryotic and fungal fractions and between 400 and 500 nt for the protozoan fraction, to better filter out non-specific amplicons. Filtered reads were clustered into zero-radius operational taxonomic units (zOTU) by USEARCH (v. 11.0.667) (Edgar, 2016) to merge reads putatively coming from the same species and retaining only those supported by 5 or more reads. Downstream analyses were performed in QIIME 1.9.0 suite (Caporaso et al., 2010). Taxonomic assignment of zOTU was performed by RDP classifier (Wang et al., 2007) against SILVA 138 database (Quast et al., 2013) using 0.8 as the confidence threshold for bacteria/archaea 16S and protozoa 18S amplicons, and against UNITE 8.3 (Abarenkov et al., 2021) for fungal ITS amplicons.

Since ITS sequences are highly variable, we did not perform the multiple alignment of the fungal zOTU and the phylogenetic tree reconstruction. Thus, for the fungal dataset, for both the alpha- and beta-diversity measures, only non-phylogenetic metrics were used. All samples were normalized to the least sequenced sample for each dataset. Alpha-diversity analysis was performed using several metrics (i.e., Shannon's diversity, chao1 diversity index, observed species, plus Faith's phylogenetic diversity index for prokaryotes and protozoa only). A non-parametric permutation-based *t*-test, with 999 random permutations, was employed to assess statistical differences. Beta-diversity analysis was based on unweighted and weighted UniFrac distances (Lozupone et al., 2011), for prokaryotes and protozoa and on Euclidean, Bray-Curtis, and Jaccard distances for fungi; the “Adonis” test function (Permutational Multivariate Analysis of Variance Using Distance Matrices, using pseudo-F ratios) using 999 random permutations was used to define significant differences among the experimental groups. Distance distribution analyses were performed by pairwise comparison between samples based on the unweighted UniFrac distance (prokaryotes and protozoa) and the Bray-Curtis distance (fungi), considering only goats with all 4 experimental points (*N* = 4). The statistical analysis was performed using the mixed procedure of SAS, version 9.4 (SAS Institute Inc.) for all biological hierarchical classes considering only groups with relative abundance >0.5% in at least one treatment. The data were analyzed with the following model:


$$\begin{array}{l} {Y_{ij}}_{\left(\text{k}\right)\text{m}}=\mu +S_{m}+G_{im}+P_{jm}+T_{\left(\text{k}\right)}+\;e_{ijm} \end{array}$$

where *Y*__*ij*_*(k)m*_ represents the dependent variable; μ is the overall mean; *S*_*m*_ represents the fixed effect of the experimental square, with *m* = 1, 2; *G*_*im*_ represents the random effect of goat *i* within the square, with *i* = 1, …, 4; *P*_*jm*_ represents the fixed effect of period *j*, with *j* = 1, …, 4 within the experimental square; *T*_(k)_ represents the fixed effect of treatment *k*, with *k* = 1, …, 4; and *e*_*ijm*_ represents the residual error. Initially, the treatment-by-period interaction was included in the statistical model; however, due to the lack of statistical significance, it was subsequently removed from the final model. Estimates of the least squares means are reported. Differences between treatments were determined using the PDIFF option. The same mixed model was used to obtain linear and quadratic polynomial contrasts. The data was log-transformed before statistical analysis. The log-transformed data were tested for normality of the residuals by using the Shapiro-Wilk test. Correlations were calculated as Spearman's rank correlation between each zootechnical parameter obtained from the experimental trial with respiration chambers and reported in the dedicated paper of Battelli et al. (2024), and the main taxa for bacteria, fungi and protozoa. Significance was declared at *P* < 0.05 and trends at 0.05 ≤ *P* < 0.10 for all the statistical analyses.

## 3 Results

### 3.1 Microbiota sequencing results

The feeding trial generated 26 rumen fluid samples. The sequencing of the microbial communities, after quality filtering and zOTU creation, generated an average of 57,629 ± 15,805, 65,282 ± 29,774, and 116,619 ± 46,859 reads for the rumen bacterial/archaeal, fungal, and protozoan communities, respectively, corresponding to an average of 43%−63% of the initial dataset. Full statistics about sequencing and zOTU creation performances are reported in Supplementary Table S1. The analysis of the rarefaction curves for both the Faith's phylogenetic diversity (PD whole tree) (prokaryotes and protozoa) and the observed species (fungi) metrics showed that most of the samples tended to reach a plateau, thus suggesting that the depth of coverage was sufficient to describe the biological diversity within the samples (Supplementary Figure S1).

For the ruminal fungal microbiota, 12 out of 26 rumen samples produced >10,000 reads and could be considered for analysis. These included 2 goats for diet C, 4 goats for diet Q2, 4 goats for diet Q4, and 2 goats for diet Q6. For the ruminal bacterial, archaeal, and protozoal microbiota, 26 out of 26 rumen samples produced >20,000 reads and could be considered for analysis. These included 6 goats for diet C, 7 goats for diet Q2, 7 goats for diet Q4, and 6 goats for diet Q6.

### 3.2 Taxonomic composition of the ruminal prokaryotic and eukaryotic microbiota

The dominant bacterial phyla identified in the rumen were Bacteroidota and Firmicutes, accounting for 40.7% and 31.4%, respectively, followed by Verrucomicrobiota (7.2%), Patescibacteria (5.2%), and Proteobacteria (5.2%). The archeal phyla Euryarcheota accounted for 4.6% on average.

**Figure 1:**
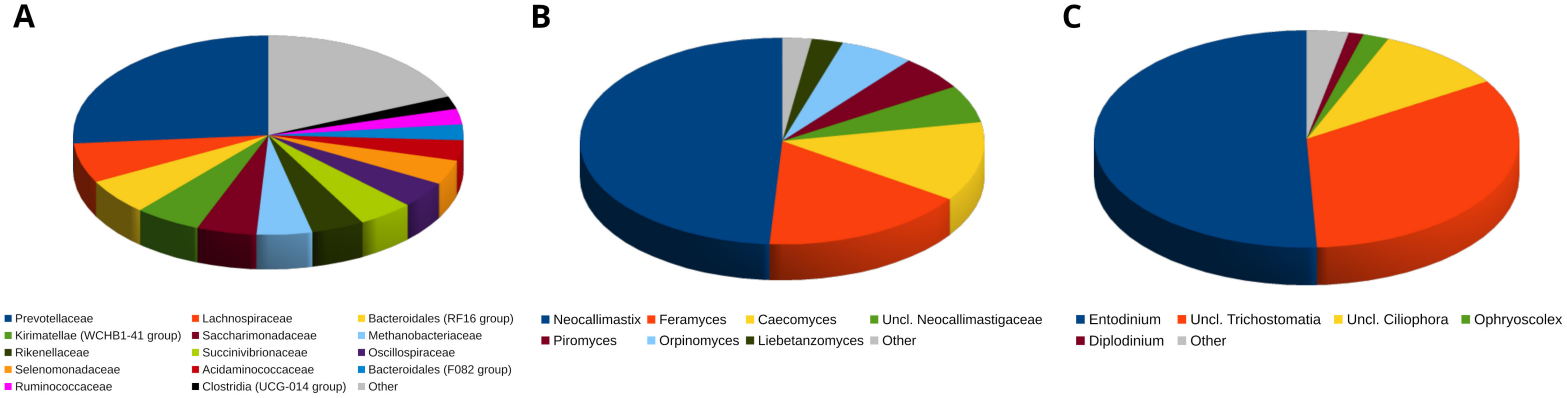
Piecharts illustrating the relative abundance over all the rumen microbiota samples at the lowest resolved taxonomic level. **(A)**: prokaryotic microbiota. **(B)**: fungal microbiota. **(C)**: protozoan microbiota. Taxa with a relative abundance <2% on average for Prokaryotes and Fungi or <1% on average for Protozoa are grouped in the “Other” category.

The lowest well-resolved taxonomy level for the rumen prokaryotic microbiota was that of families, whereas at the genus level many assignments were “uncultured bacterium” or “unclassified”. The most abundant family was Prevotellaceae, accounting for about 26.3% of the relative abundance, on average, followed by Lachnospiraceae (6.4%), Bacteroidales RF16 group (5.8%), Kirimatellae WCHB1-41 group (5.5%), Saccharimonadaceae (5.0%), Rikenellaceae (4.4%), Succinivibrionaceae (4.6%) and Oscillospiraceae (4.3%), while the dominant archaeal family was Methanobacteriaceae, summing up to 4.6% of the relative abundance on average. Globally, the 20 most abundant families accounted for >89% of the total relative abundance. Based on the available data, the fungal ruminal microbiota was dominated by Neocallimastigomycota (97.6% on average), followed by Ascomycota (1.0%). The most abundant genus was *Neocallimastix*, accounting for an average 49.0% of the total relative abundance, followed by *Feramyces* and *Caecomyces*. These three most abundant genera accounted for ~78% of the total relative abundance. The ruminal protozoan microbiota showed little differentiation at high taxonomic levels and a consistent part of it (44% on average) was undefined at the genus level. Based on the assigned genera, the protozoan microbiota was dominated by *Entodinium* (50.8%), followed by unclassified members of *Trichostomatia* (33.1%), and *Ciliophora* (9.8%). Together, these taxa represented >93% of the total relative abundance. The taxonomic composition of bacteria/archaea, fungi and protozoa in the rumen according to the dietary treatment is detailed in Figure 1 and in Supplementary Figure S2.

### 3.3. Biodiversity and composition of the ruminal microbiota in the different experimental diets

**Figure 2:**
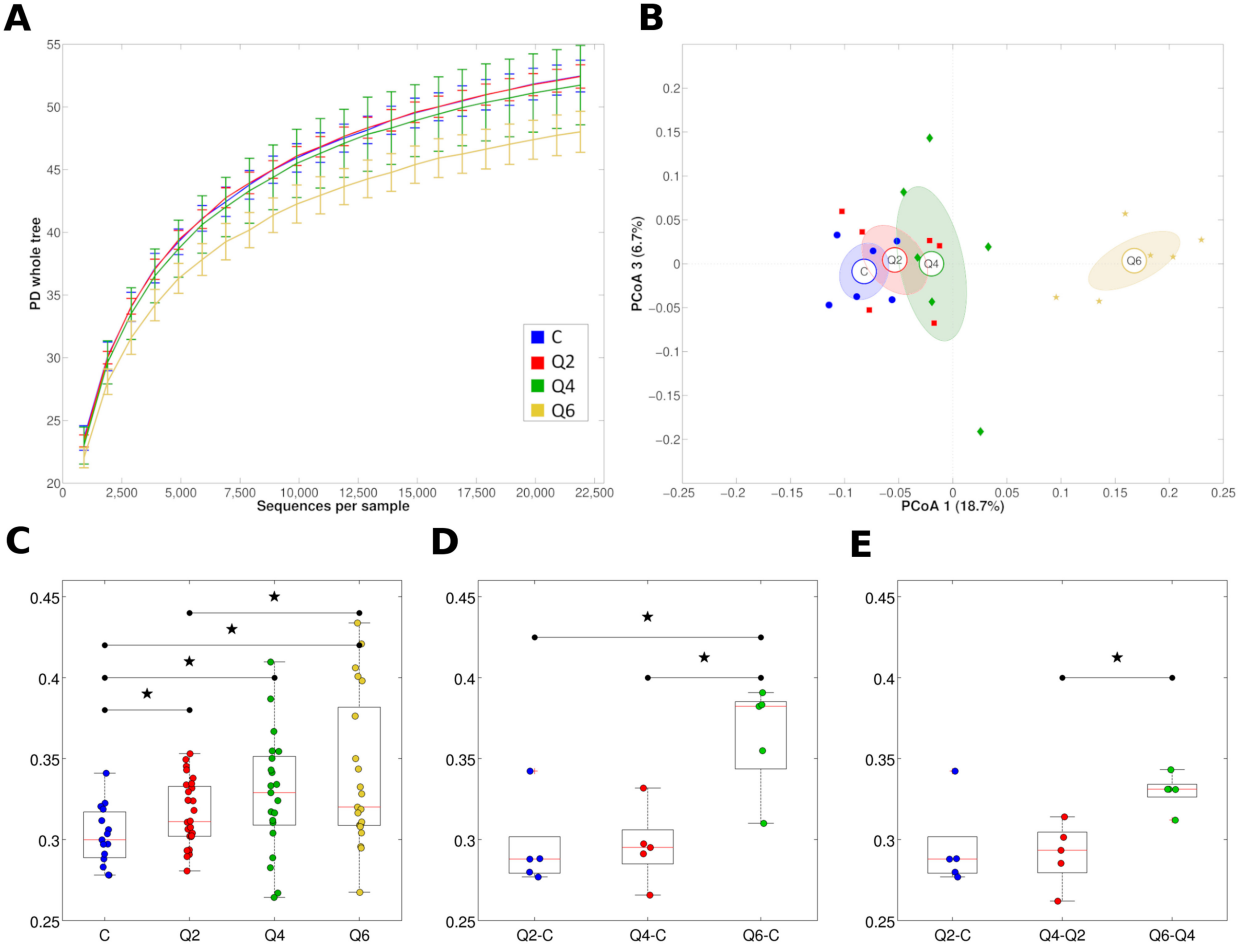
**(A)** Rarefaction curves of the ruminal prokaryotic microbiota according to the PD whole tree metrics based on the zero-radius operational taxonomic units (zOTU) tree for all the experimental diets assessed in this study. The line represents the average over the samples collected from goats subjected to the same diet; error bars representing the intra-diet standard deviation are also represented. **(B)** principal coordinate analysis (PCoA) based on the unweighted UniFrac distance between samples illustrating the clustering of rumen microbiota samples according to goat diet. Each point represents a sample, colored according to the diet group, centroids are the average of the coordinates and ellipses represent the SEM-based confidence interval. The first and third principal coordinates are represented. **(C)** Boxplots representing the distribution of the unweighted UniFrac distances among samples from the same diet group. Stars above boxplots indicate a statistically significant difference (*P* < 0.05) between groups. **(D)** Boxplots representing the distribution of the unweighted UniFrac distances among samples from the three diet groups (i.e., Q2, Q4, and Q6) and C diet. Stars above boxplots indicate a statistically significant difference (*P* < 0.05) between groups. **(E)** Boxplots representing the distribution of the unweighted UniFrac distances among samples for increasing tannin concentrations. Stars above boxplots indicate a statistically significant difference (*P* < 0.05) between groups. C: control diet. Q2, Q4, Q6: diets integrated with 2, 4, 6% on DM of quebracho tannin extract, respectively.

The Q6 diet was associated with a significantly lower biodiversity (alpha-diversity) of the rumen prokaryotic microbiota compared to both C and Q2 (*P* = 0.030 and 0.012, respectively), while Q2 and Q4 did not show statistically significant differences between them or compared to the C diet (Figure 2A). The alpha-diversity of the fungal and protozoan rumen microbiota did not change significantly for the different diets or animals, although there was a tendency toward increased biodiversity of the fungal microbiota in Q4 and Q6 (Supplementary Figure S3, panels A and B). In terms of microbial composition, the rumen samples of the Q6 diet were significantly different from those of the other diets (*P* < 0.006 for all pairwise comparisons among the experimental groups, unweighted UniFrac distance; *P* = 0.013 for Q6 vs. C, weighted UniFrac distance), while Q2 and Q4 did not show statistically significant differences with C or between them. By Principal Coordinate Analysis (PCoA), the samples corresponding to the same experimental diet clustered together and showed a trend in the first principal coordinate according to increasing CT concentration; Q6 showed a clear separation from all the others (Figure 2B).

The rumen microbiota composition changed significantly for Q6 (*P* < 0.013) compared to C also according to the weighted UniFrac metric. The unweighted UniFrac phylogenetic distance among rumen samples within the same diet (regardless of CT concentration) was higher than the distance among rumen samples within the C diet (*P* = 0.040, *P* = 0.017 and *P* = 0.005, for Q2, Q4, and Q6, respectively), indicating that the CT-integrated diets created more dispersed samples in terms of microbiota diversity; the rumen beta-diversity of goats increased with increasing CT concentrations (Figure 2C). The phylogenetic distance also increased with increasing CT concentrations; that is, the distance between Q6 and C was higher than the distance between Q2/Q4 and C (*P* = 0.016 for both comparisons), suggesting more diverse prokaryotic microbiota profiles with increasing dietary CT concentrations (Figure 2D). Finally, the unweighted UniFrac distance between Q6 and Q4 was significantly higher than the distance between Q4 and Q2, suggesting that the highest CT concentration (i.e., Q6) is the one that induces the most significant shifts in the prokaryotic microbiota composition (Figure 2E). Beta-diversities for both fungi and protozoa based on the diet were not statistically significant (Supplementary Figure S3, panels C and D).

Interestingly, the prokaryotic, fungal and protozoan microbial composition all showed a goat-specific fingerprint, since the distances among samples coming from the same animal, regardless of the diet, were lower than those between samples from different goats (prokaryota: *P* = 0.006, unweighted UniFrac; fungi: *P* = 0.009, Bray-Curtis; protozoa: *P* = 0.004, unweighted UniFrac).

### 3.4 Changes in the relative abundance of taxonomic groups in the ruminal microbiota according to dietary treatment

**Figure 3:**
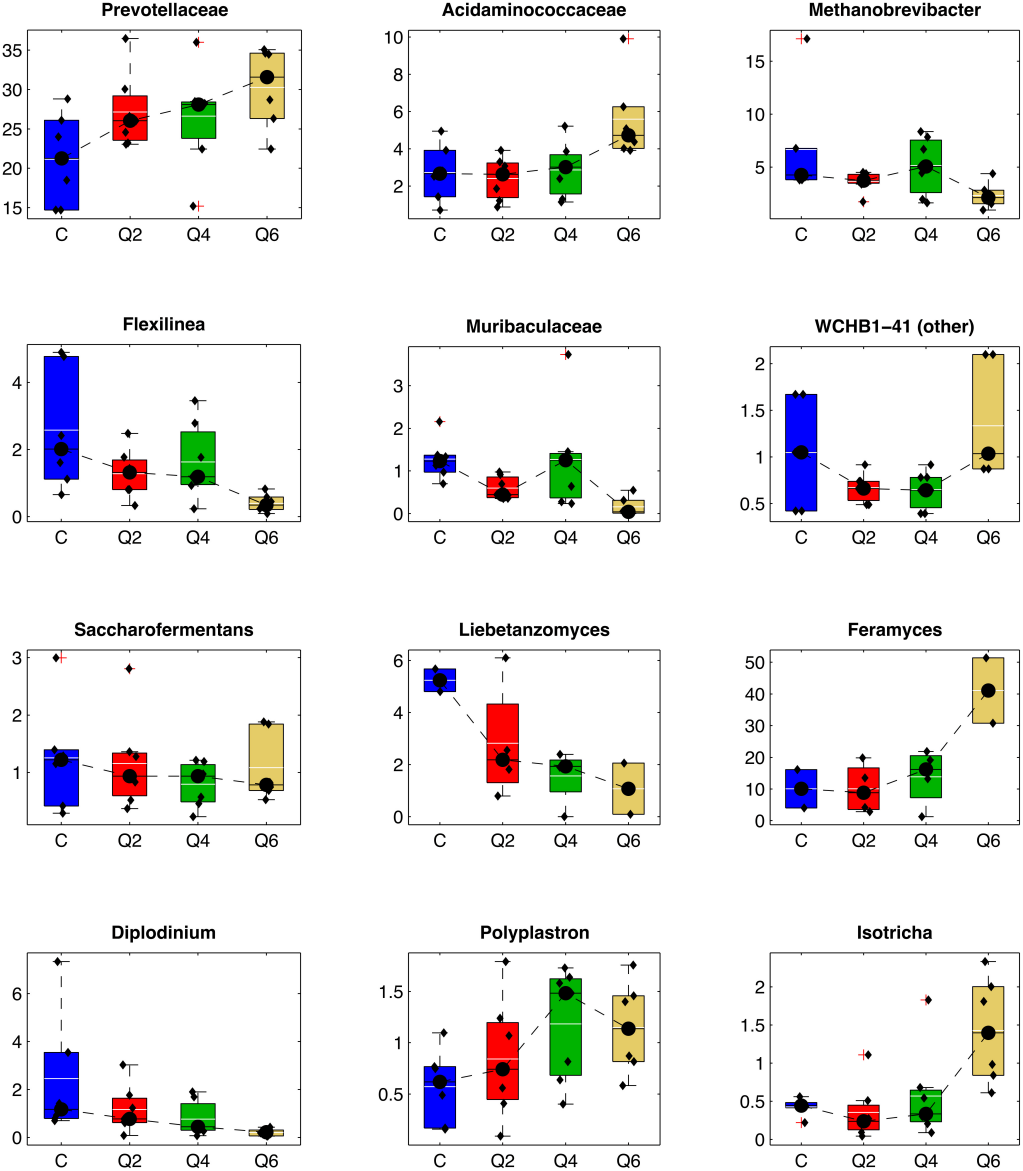
Boxplot representing the relative abundance of some selected taxa (average rel. ab >1% in at least one treatment). In each boxplot, individual samples are represented as black dots; average abundance is represented as a white line, whereas median abundance is depicted as a black line. C: control diet. Q2, Q4, Q6: diets integrated with 2, 4, 6% on DM of quebracho tannin extract, respectively.

To understand the specific changes occurring upon dietary integration with quebracho CT, we compared the relative mean abundance of the different prokaryotic and eukaryotic microbial taxa between Q2, Q4, and Q6 and the C diet, respectively (Figure 3 and Supplementary Tables S2–S8). Considering the prokaryotic community, at the phylum level, the integration with CT induced a significant depletion of Firmicutes for Q2 and Q4 with a quadratic effect, and a corresponding trend toward the increase of ruminal Bacteroidota, leading to an inversion of the Firmicutes/Bacteroidota ratio for all the diets with CT inclusion (Q2, Q4, and Q6) when compared to C. The introduction of increasing concentration of CT in the diet resulted in a linear increase in the relative abundances of members of the families Prevotellaceae (together with the related genera Prevotellaceae UCG-001, Prevotella_9, and Prevotellaceae UCG-004), and Acidaminococcaceae (*Succiniclasticum*). The abundance of many taxa decreased, including the archaea *Methanobrevibacter* (with the family Methanobacteriaceae), and the bacteria *Flexilinea* (with the associated phylum, Chloroflexi, and family, Anaerolinaeceae), *Fretibacterium* (with the associated phylum, Synergestota, and family, Synergistaceae), the Pirellulaceae p-1088-a5 gut group, Lachnospiraceae XPB1014, and the families of Oscillospirales UCG-011 and Muribaculaceae.

At the same time, increasing CT concentrations resulted also in quadratic effects on the archaea Methanomethylophilaceae, and members of the WCHB1-41 order, which increased at Q2 and Q4 while decreasing at Q6, and on Erysipelotrichaceae, *Saccharofermentans* (and its family Hungateiclostridicaece), *Eubacterium coprostanoligenes* group, *Butyrivibrio* and Lachnospiraceae NK3A20, which, on the other hand, decreased at Q2 and Q4.

For the fungal community, the relative abundance of genus *Liebetanzomyces* showed a trend toward a linear decrease at increasing CT concentrations, whereas the phylum Ascomycota and the genera *Feramyces* and *Aspergillus* had the opposite trend.

Among protozoa the genus *Diplodinium* decreased, while *Polyplastron* increased linearly with CT concentration and *Isotrichia* had a quadratic behavior (i.e., reduced at Q2 and Q4, increased at Q6).

### 3.5 Correlations of ruminal taxa relative abundance with zootechnical parameters

**Figure 4:**
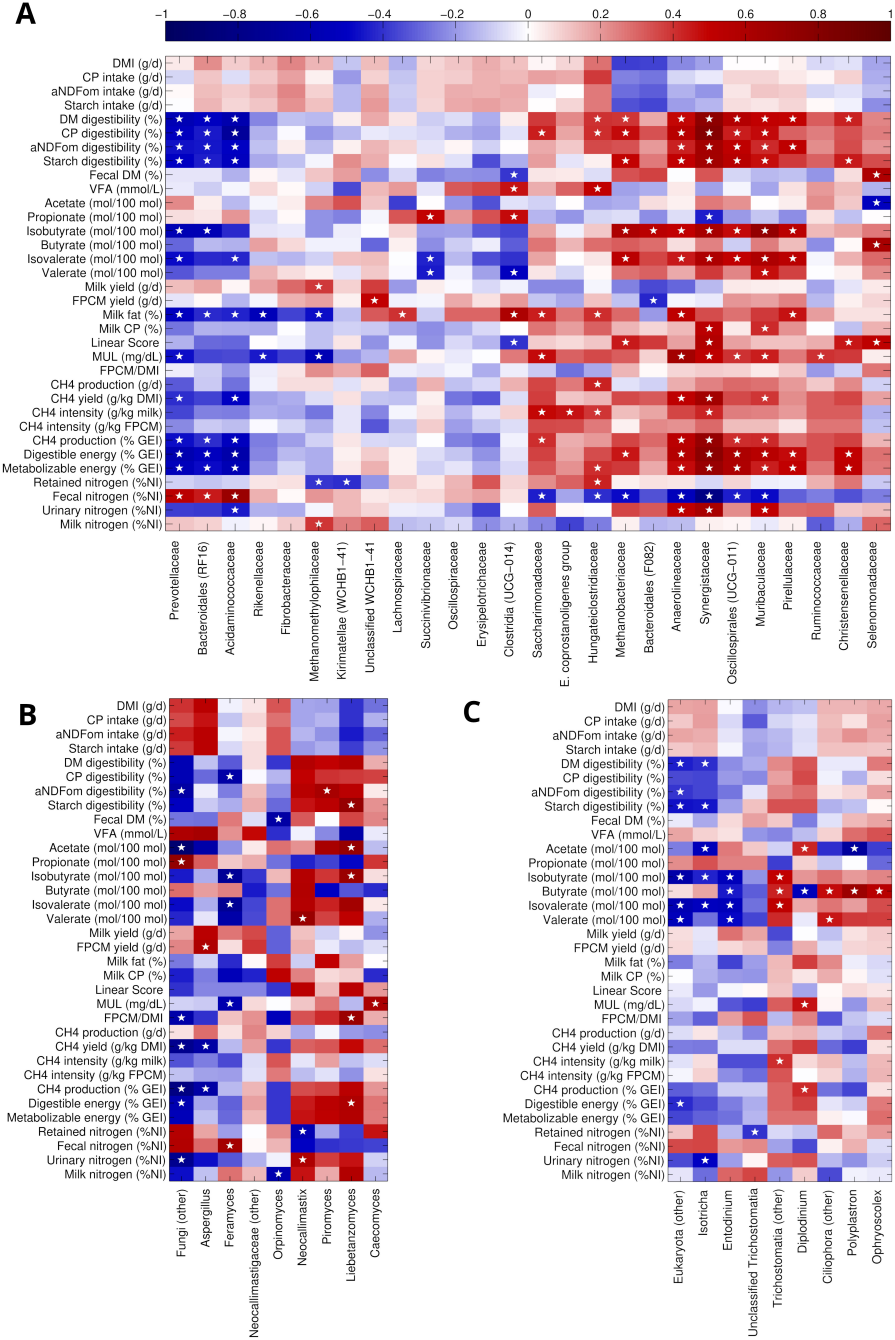
Spearman's rank correlation between zootechnical parameters and the main prokaryotic and eukaryotic taxa. For prokaryota **(A)**, we considered the classification at the family level and only families with mean relative abundance >0.5%; for fungi **(B)** and protozoa **(C)**, we considered genera with a mean relative abundance of 0.2% or more. White stars indicate a significant (hypothesis of no correlation against the alternative hypothesis of a non-zero correlation, *P* < 0.05) taxon-parameter combination. Heatmaps were clustered over taxa according to Euclidean distance and complete linkage to highlight common patterns of correlation for multiple taxa.

A group of taxa (i.e., Methanobacteriaceae, Anaerolinaceae, Synergestaceae, Oscillospirales UCG-011, Muribaculaceae, Pirellulalceae), whose abundance was linearly decreasing with increasing CT concentration (Supplementary Table S5), was negatively correlated to fecal nitrogen excretion (% of nitrogen intake) and positively correlated to several parameters such as (Figure 4A): DM, CP, α-amylase- and sodium sulfite-treated NDF corrected for insoluble ash (aNDFom), and starch digestibility, methane production (g CH_4_/d) and yield (g CH_4_/kg DMI), isobutyrate and isovalerate concentration (mol/100 mol VFA), urinary nitrogen excretion, digestible and metabolizable energy (% of gross energy intake). Similarly, all these families but Metahnobacteriaceae were also positively correlated to milk urea level (MUL), milk fat and milk CP. On the other hand, Prevotellaceae, Bacteroidales RF-16 group and Acidaminoccoaceae tended to increase with CT concentration and were positively correlated to fecal nitrogen and negatively to all the parameters described above. Among the other taxa significantly altered with the diet, Methanomethylphilaceae behaved similarly to Prevotellaceae, also showing a negative correlation to retained nitrogen and a positive one to milk nitrogen excretion and milk yield; Hungateiclostridiaceae, on the other hand, was more similar to the group including Methanobacteriaceae, also revealing a positive correlation to VFA and retained nitrogen; finally, Unclassified WCHB1-41 showed a direct correlation to fat and protein corrected milk (FPCM) yield.

Within the fungal community, *Liebetanzomyces, Neocallimastix* and *Piromyces* had a trend toward a reduction with increasing CT concentration in the diet (Supplementary Table S7) and were positively correlated to a vast group of parameters (Figure 4B) (i.e., DM, CP, aNDFom, and starch digestibility, acetate, isobutyrate, isovalerate, and valerate concentrations, FPCM/DMI, methane production and yield, digestible and metabolizable energy, urinary and milk nitrogen excretion). *Feramyces*, on the other hand, increased with Q6 diet and showed positive correlation to fecal nitrogen excretion, and negative correlations to: CP digestibility, isobutyrate, isovalerate, and valerate concentrations, and MUL. In general, only a few correlations in the fungal dataset were statistically significant, since many experimental points were missing.

For protozoa (Figure 4C), *Polyplastron* (increased with increasing CT concentration—Supplementary Table S8), was positively correlated to butyrate and inversely to acetate; *Diplodinium* (decreased with increasing CT concentration) was positively correlated to acetate concentration, MUL, and methane production, and negatively to butyrate; *Isotrichia* (increased with increasing CT concentration) was negatively correlated to DM and starch digestibility, acetate, isobutyrate, isovalerate concentrations, and urinary nitrogen excretion.

## 4 Discussion

Supplementation with CT is suggested as a dietary strategy for reducing methane production in ruminants. A previous study (Battelli et al., 2024) assessed three different levels of dietary quebracho CT extract integration in comparison with a standard diet without CT. Methane emission measurement in open circuit respiration chambers revealed that quebracho CT was indeed able to lower methane emissions, although with a negative impact on feed digestibility and feed efficiency. The methane yield (g CH_4_/kg DMI) and the methane intensity expressed per kg of milk (g CH_4_/kg milk) were linearly reduced by CT inclusion, but not methane intensity expressed per kg of FPCM. Moreover, the methane production per kg of digestible aNDFom was linearly increased by CT, due to the greater reduction effect of feed digestibility compared to the anti-methanogenic effect of CT. Here, we investigated the ruminal microbiota to understand the impact of CT on prokaryotic and eukaryotic diversity, and to draw some insights into their relationships with methane production and nutrient utilization.

When considering all the ruminal samples collected during the study, independently from the dietary group, the bacterial phyla Bacteroidota and Firmicutes dominated the rumen prokaryotic microbiota, with a high abundance of the family Prevotellaceae, followed by Lachnospiraceae, consistent with previous reports (Zened et al., 2013; Belanche et al., 2014; Cremonesi et al., 2018). Goats have a higher relative abundance of Veillonellaceae (Henderson et al., 2015) and the relative abundance of Fibrobacterota is lower than in cows (Giger-Reverdin et al., 2020), as also seen in the present study. Archaea accounted for about 5% and the family Methanobacteriaceae represented the largest share with 4.6%, in agreement with the literature reporting a similar composition for the goat rumen microbiota (Nelson et al., 2010; Belanche et al., 2014; Paul et al., 2015; Zhang et al., 2022).

The integration of feeds with quebracho CT reduced the biodiversity of the prokaryotic goat rumen microbiota, in line with previous studies on cows (Díaz Carrasco et al., 2017; Cidrini et al., 2022). The extent of biodiversity reduction followed CT concentration in the diet and was statistically significant at Q6, indicating that high quebracho CT concentrations are required to induce compositional shifts in the rumen microbiota.

Numerous ruminal bacterial and archaeal taxa were modified by dietary quebracho CT inclusion. At the phylum level, the most evident change was a decrease in Firmicutes accompanied by a trend toward enrichment in Bacteroidota. This led to an inversion in the Firmicutes/Bacteroidota ratio in all CT-integrated diets when compared to the C diet, in which Firmicutes predominated. Jakhesara et al. (2010) and Rabee et al. (2024) also reported an enrichment of Bacteroidota in the rumen of tannin-challenged goats. A preliminary metagenomic study on two different goat lineages, Repartida and Canindè, observed that the Firmicutes/Bacteroidota ratio was inverted in both genetic groups when CT were included in the feed (Silva de Sant'ana et al., 2022). These two phyla are responsible for most of the rumen fermentation of structural and non-structural carbohydrates. According to White et al. (2014), Firmicutes and Bacteroidota degrade plant biomass differently: Firmicutes break down cell surfaces, whereas Bacteroidota mostly degrade intracellular and periplasmic materials. El Kaoutari et al. (2013) found that, on average, Firmicutes encode fewer glycan-cleaving enzymes than Bacteroidota in human gut microbiota, suggesting that an increase in the Firmicutes/Bacteroidota ratio in may impair dietary fiber utilization in the rumen. As previously reported (Battelli et al., 2024), aNDFom digestibility decreased linearly with quebracho CT inclusion, from 58.0% in C to 44.6% in Q6. The inversion of the Firmicutes/Bacteroidota ratio is likely driven by the formation of CT-feed complexes, which limit substrate availability for Firmicutes, particularly cellulolytic families like Ruminococcaceae, by binding to plant cell walls (Naumann et al., 2017; Liu et al., 2018; Zhang et al., 2025). In contrast, Bacteroidota, such as Prevotella, tolerate tannins and degrade a broader range of substrates, resulting in their relative increase (Nelson et al., 1997; Li et al., 2013), as seen in studies on goats (Wang et al., 2022; Rabee et al., 2024) and bovines (Díaz Carrasco et al., 2017). While direct antimicrobial effects of CT may contribute, the primary mechanism appears an altered substrate availability through the formation of CT-feed complexes. Although our study cites evidence for these complexes in rumen dynamics, we did not evaluate their effects using binding inhibitors like PEG (Fagundes et al., 2020). Such experiments could provide additional mechanistic insights by isolating CT-specific effects on goat microbiota and future research should incorporate these approaches to further clarify CT-driven microbial shifts.

An increase in Prevotellaceae (phylum Bacteroidota) was observed with quebracho CT supplementation. Prevotellaceae are core hemicellulose degraders and include the well-known rumen bacterium *Prevotella ruminicola* (Moraïs and Mizrahi, 2019a). Moreover, the genus *Prevotella* can tolerate tannins (Nelson et al., 1997; Li et al., 2013). In an *in vitro* study on cow ruminal fluid with similar tannin concentrations (0%, 2%, 4%, 6%, 8% of both CT and HT on an as-fed basis), the percentage of *Prevotella* was linearly increased by CT inclusion (Battelli et al., 2023). Silva de Sant'ana et al. (2022) also reported an increase in the relative abundance of *Prevotella* when CT were included in the diet of lactating goats. By degrading hemicellulose, *Prevotella* produce succinate, acetate, formate, lactate, and propionate (Mizrahi et al., 2021). Chemical inhibition of methanogenesis in goats led to an adaptation to higher hydrogen levels by shifting fermentation to propionate, as also observed by Battelli et al. (2024) for the Q6 diet, likely mediated by an increase in the population of hydrogen-consuming *Prevotella* and *Selenomonas* spp. (Denman et al., 2015). The genus *Succiniclasticum* and the related family Acidaminococcaceae were also increased by the highest CT inclusion. This was in agreement with the results of Silva de Sant'ana et al. (2022) who suggested a tolerance of Acidaminococcaceae family to CT presence. Moreover, bacteria within this family ferment succinate to propionate in agreement with the highest proportion of propionate found with Q6 diet (Battelli et al., 2024). *Succiniclasticum ruminis* is a key microbial member that converts lactate and succinate into VFA (Mizrahi et al., 2021). Accordingly, the above changes in the rumen microbial makeup may also lead to the generation of higher amounts of VFA (Moraïs and Mizrahi, 2019b). We did not observe statistically significant changes in total VFA levels, probably due to the reduction in diet digestibility (Battelli et al., 2024); however, the proportion of some VFA including propionate (increased with Q6), isobutyrate and isovalerate (both decreased) changed significantly. As the latter two are produced by deamination, their decrease might be related to lower rumen protein degradability and decrease in proteolytic bacteria. Accordingly, we found that Anaerolineaceae, Synergistaceae, Oscillospirales (UCG-011 group), and Muribaculaceae, all decreasing for increasing CT concentrations, had a positive correlation with protein digestibility and the concentrations of isobutyrate and isovalerate, whereas Prevotellaceae, Bacteroidales (RF16 group), and Acidaminococcaceae were negatively correlated with these variables. McSweeney et al. isolated proteolytic and peptidolytic bacteria from the rumen of sheep and goats fed CT from the shrub of *Calliandra calothyrsus*, but observed that the isolated bacteria were not able to digest protein complexed with CT (McSweeney et al., 1999). In another study, Min et al. observed *in vitro* that the proteolytic activities and the growth of selected proteolytic rumen bacteria were inhibited by CT from *Lotus corniculatus* (Min et al., 2002).

Most interestingly, all the levels of quebracho CT integration induced a decrease in the relative abundance of *Methanobrevibacter*, within the family Methanobacteriaceae. This is particularly relevant as the family Methanobacteriaceae produce methane as the main catabolic product (Mizrahi et al., 2021). The genus *Methanobrevibacter* is the most dominant, accounting for up to 70% of the rumen archaeal community (Friedman et al., 2017). Similarly, *Flexilinea* (family Synergistaceae), decreasing with CT concentration, has been described as a fiber-digesting bacteria, reported to degrade different kinds of carbohydrates and its growth is enhanced in the presence of methanogens (Sun et al., 2016). *Fretibacterium* has been reported to have an amylolytic activity and to be involved in biohydrogenation of linoleic acid (C18:2n6c) in goat rumen (Mavrommatis et al., 2021). On the contrary, Methanomethylophilaceae were significantly increased with CT integration. The known members of this family reduce methanol, monomethylamine, dimethylamine, and trimethylamine into methane, with hydrogen as the electron donor (Borrel et al., 2023). The lower abundance of Methanobacteriaceae may have created a more favorable environment for Methanomethylophilaceae, likely due to the increased availability of hydrogen in the rumen. The observed decrease in methane emissions with CT supplementation can be linked to changes in hydrogen metabolism and the methanogenic community structure. The increase in propionate production, a hydrogen sink, likely reduced the availability of hydrogen for methanogenesis (Battelli et al., 2024). Additionally, CT may directly inhibit dominant hydrogenotrophic methanogens like *Methanobrevibacter*, as shown by their reduced relative abundance (Zhu et al., 2025). Concurrently, the rise in Methanomethylophilaceae, which use methyl compounds and hydrogen, suggests a shift in the methanogenic pathway. Despite this shift, total methane production decreased, suggesting that the reduction in hydrogen availability and the inhibition of key methanogenic taxa outweighed any potential increase from methyl-reducing methanogens. Therefore, quebracho CT supplementation may indeed influence methane production also in goats by altering methanogenic taxa, but it also impacts feed conversion efficiency (Battelli et al., 2024). High CT concentrations are needed for significant effects on the ruminal relative abundance of these taxa. Longer feeding periods may be allow lower CT concentrations to be effective, although adaptation phenomena may also develop. This highlights the complex interplay between dietary additives, microbial community dynamics, and rumen fermentation pathways in mitigating enteric methane emissions.

The rumen microbiota consists primarily of bacteria (about 95%) with archaea (2–5%), eukaryotic protozoa and fungi (0.1–1% of the total microbial richness) playing crucial metabolic roles in spite of their low abundance (Jami et al., 2013; Moraïs and Mizrahi, 2019a). We observed a significant decrease in the anaerobic fungal genus *Liebetanzomyces* and the corresponding family Neocallimastigaceae starting from the lowest-CT integration (Q2). *Liebetanzomyces* are slow-growing anaerobic fungi that have been discovered in the goat rumen, where they play a pivotal role in the degradation of lignocellulosic feed (Gruninger et al., 2014; Joshi et al., 2018). In ruminant gastrointestinal tract, Neocallimastigomycota are reported to be responsible for fermenting 18–63% of untreated plant biomass despite comprising only about 8% of the total microbial biomass (Gruninger et al., 2014). Their reduction is in line with decreased aNDFom digestibility due to CT supplementation, likely because CT limit substrate access (Battelli et al., 2024; Naumann et al., 2017). Main methanogens associated with anaerobic fungi, including *Methanobrevibacter, Methanobacterium*, and *Methanosphaera* have been identified as the main methanogens associated with anaerobic fungi (Leis et al., 2014; Joshi et al., 2018), in line with our observations concerning the decrease in *Methanobrevibacter*. *Feramyces*, a relatively novel fungal genus able to grow on sugars and plant biomass, as well as to metabolize a wide range of mono-, oligo-, and polysaccharides (Hanafy et al., 2018), increased with dietary CT concentration. *Aspergillus*, a known tannin degrader, also increased, potentially mitigating CT effects by breaking down tannins, as observed on the liquid surfaces of tannery pits and tannery wastes. *Aspergillus* easily develops on the surface of tannin-rich woods including quebracho (Bhat et al., 1998).

The fungal community shifts have significant implications for rumen function. The decrease in the relative abundance of *Liebetanzomyces* and *Neocallimastigaceae* likely contributed to reduced fiber degradation, while *Feramyces* and *Aspergillus* increases indicate adaptive responses to CT-induced substrate changes (Gruninger et al., 2014). Recent studies suggest that anaerobic fungi like *Neocallimastigacee* also produce hydrogen, influencing methanogenesis, which may explain their link to methanogen reduction (Ma et al., 2022). However, methodological challenges, such as limited sequencing depth for low-abundance fungi, may restrict detailed analysis, requiring advanced techniques like targeted metagenomics in future research (Edwards et al., 2019). In this respect, it should be highlighted that in our study fungal microbiota investigations were carried out on a reduced number of samples compared to prokaryotic microbiota investigations (12 vs. 26, respectively).

The rumen protozoal community showed varied responses to CT supplementation. The highly abundant genus *Entodinium* was not affected by CT, indicating maintained core fermentation processes, even at high dietary levels. In contrast, *Diplodinium*, a fibrolytic genus, decreased significantly, in line with reduced aNDFom digestibility due to CT-feed complexes (Takenaka et al., 2004; Naumann et al., 2017; Battelli et al., 2024). Diplodiniiae ciliates, including *Diplodinium*, contribute substantially to rumen fiber digestion, and their reduction likely reflects tannin-related substrate limitations (Martinele et al., 2010). Conversely, *Polyplastron* and *Isotrichia* increased, possibly indicating shifts in fermentation toward non-fibrous feed components, as these genera are associated with carbohydrate metabolism and hydrogen production (Belanche et al., 2014). While our study thoroughly characterized the prokaryotic community, protozoal and fungal analyses face methodological challenges due to low abundance and sequencing depth limitations. Advanced techniques, such as targeted amplicon sequencing or microscopy, could provide deeper insights into protozoal interactions with CT and their contributions to rumen function (Newbold et al., 2015).

While our study demonstrated the immediate effects of quebracho CT on goat ruminal microbiota, the potential for long-term adaptation warrants further investigation. Over extended periods, the ruminal microbiota may adapt by selecting for CT-tolerant or CT-degrading microorganisms, such as *Aspergillus* or specific bacterial taxa (Odenyo et al., 1997; Goel et al., 2005). This adaptation could potentially mitigate some negative impacts on feed digestibility while maintaining methane mitigation benefits. However, given the persistent formation of CT-feed complexes, complete restoration of digestibility may not be achievable. Future research should include long-term feeding trials to investigate how ruminal microbiota adapt to sustained CT exposure, using advanced sequencing techniques to track community dynamics and functional shifts. Dose-response studies over varying durations could identify optimal CT concentrations that balance methane reduction with minimal losses in digestibility. Additionally, characterizing CT-degrading microbes and their roles in adaptation could provide insights into enhancing rumen function under tannin-rich diets. Comparative studies across ruminant species and assessments of animal performance and sustainability would further refine the application of CT as a methane mitigation strategy.

## 5 Conclusion

Increasing levels of dietary integration with CT induced increasingly relevant changes in the rumen microbiota. Quebracho CT successfully reduced the abundance of ruminal taxa responsible for methane production, indicating a shift toward a lower methane-producing microbial community state. On the other hand, as CT affected feed conversion efficiency, a negative influence of CT on substrate utilization should be taken into account as a contributing factor in the total reduction of methane emissions.

## Data Availability

Raw sequencing data for this project are available in NCBI Short-Read Archive (SRA) under accession number PRJNA1003434 (https://www.ncbi.nlm.nih.gov/bioproject/PRJNA1003434).
