# Supplementary material for: Reducing enteric methane emission in dairy goats: impact of dietary inclusions of quebracho tannin extract on ruminal microbiota
Source: Front Microbiol. 2025 Jul 7;16:1595924. doi: 10.3389/fmicb.2025.1595924 (PMC12277267; doi:10.3389/fmicb.2025.1595924)
Supplement: Supplementary file 1 [file Data_Sheet_1.pdf]

Reducing Enteric Methane Emission in Dairy Goats: Impact of dietary inclusions of Quebracho Tannin Extract on Ruminal Microbiota

P. Cremonesi[1+], M. Severgnini,[2,3+] M. Battelli[4], V. Monistero[5], M. Penati[5], A. L. Gazzonis[5,6], B. Castiglioni[1], L. Rapetti[4], M. T. Manfredi[5,6], M. F. Addis[5,7]*

[1]Institute of Agricultural Biology and Biotechnology – National Research Council (IBBA-CNR), Lodi, Italy

[2]Institute of Biomedical Technologies – National Research Council (ITB-CNR), Segrate (MI), Italy

[3]National Biodiversity Future Center S.c.a.r.l. Palermo, Italy

[4]Department of Agricultural and Environmental Sciences – Production, Landscape, Agroenergy, University of Milan, Milan, Italy

[5]Department of Veterinary Medicine and Animal Sciences, University of Milan, Lodi, Italy

[6]Research Laboratory of Animal Parasitic Diseases and Zoonoses (Parvetlab), University of Milan, Lodi, Italy

[7]Laboratory of Animal Infectious Diseases (MiLab), University of Milan, Lodi, Italy

[+]These authors contributed equally to this work and share first authorship

*Corresponding author: filippa.addis@unimi.it

TABLE OF CONTENTS

SUPPLEMENTARY FIGURES 3
Supplementary Figure S1 3
Supplementary Figure S2 4
Supplementary Figure S3 5
SUPPLEMENTARY TABLES 6
Supplementary Table S1 6
Supplementary Table S2 6
Supplementary Table S3 7
Supplementary Table S4 8
Supplementary Table S5 9
Supplementary Table S6 10
Supplementary Table S7 11
Supplementary Table S8 12

SUPPLEMENTARY FIGURES

Supplementary Figure S1. Rarefaction curves of the ruminal microbiota for all the samples of this study. Each line represents a sample; (A) Samples from rumen, prokaryota community (PD whole tree metric); (B) Samples from rumen, fungal community (observed species metric); (C) Samples from rumen, protozoan community (PD whole tree metric).

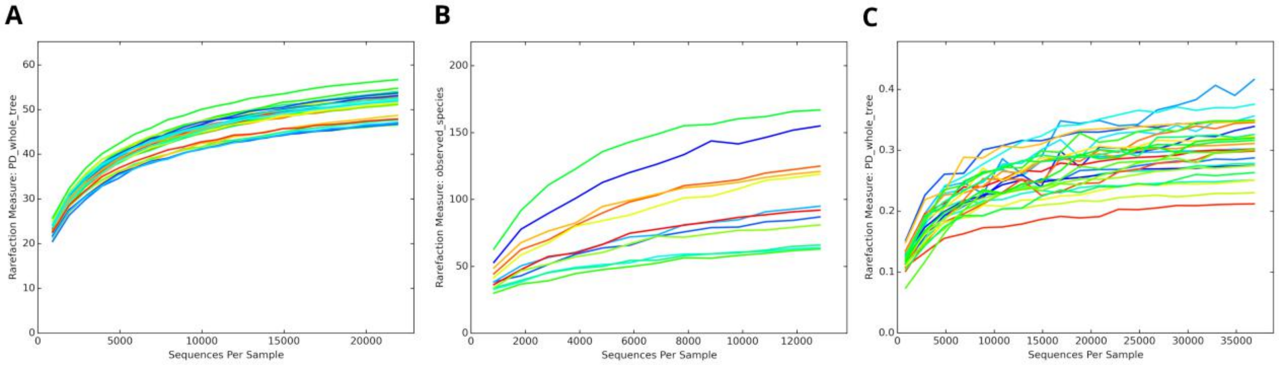

Supplementary Figure S2. Stacked bar chart illustrating the relative abundance at the lowest resolved taxonomic level in the rumen microbiota of all goats according to the dietary treatment. C: control diet. Q2, Q4, Q6: diets integrated with 2, 4, 6% on DM of quebracho tannin extract, respectively. Taxa with a relative abundance <1% on average are grouped in the "Other" category. Top (A): prokaryotic microbiota. Middle (B): fungal microbiota. Bottom (C): Protozoan microbiota.

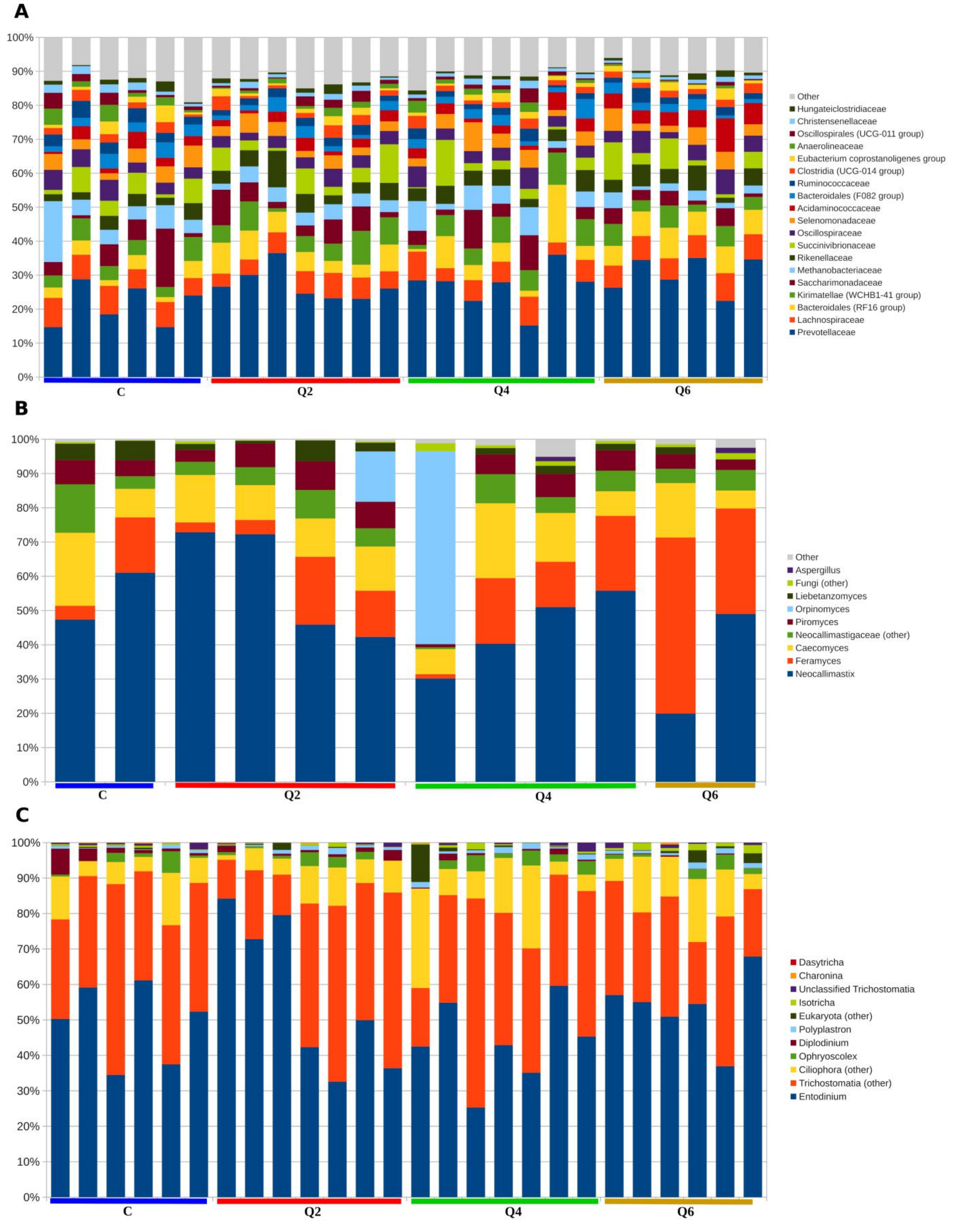

Supplementary Figure S3. Rarefaction curves of the ruminal prokaryotic microbiota according to the observed species for fungal community (A) and the PD_whole_tree metric (B) for the prozotoan community for all the experimental diets assessed in this study. The line represents the average over the samples collected from goats subjected to the same diet; error bars representing the intra-diet standard deviation are also represented. (B) principal coordinate analysis (PCoA) based on the Bray-Curtis distance for fungi (C) and the unweighted UniFrac distance for protozoa (D), illustrating the clustering of rumen microbiota samples according to goat diet. Each point represents a sample, colored according to the diet group, centroids are the average of the coordinates and ellipses represent the SEM-based confidence interval. The first and second principal coordinates are represented.

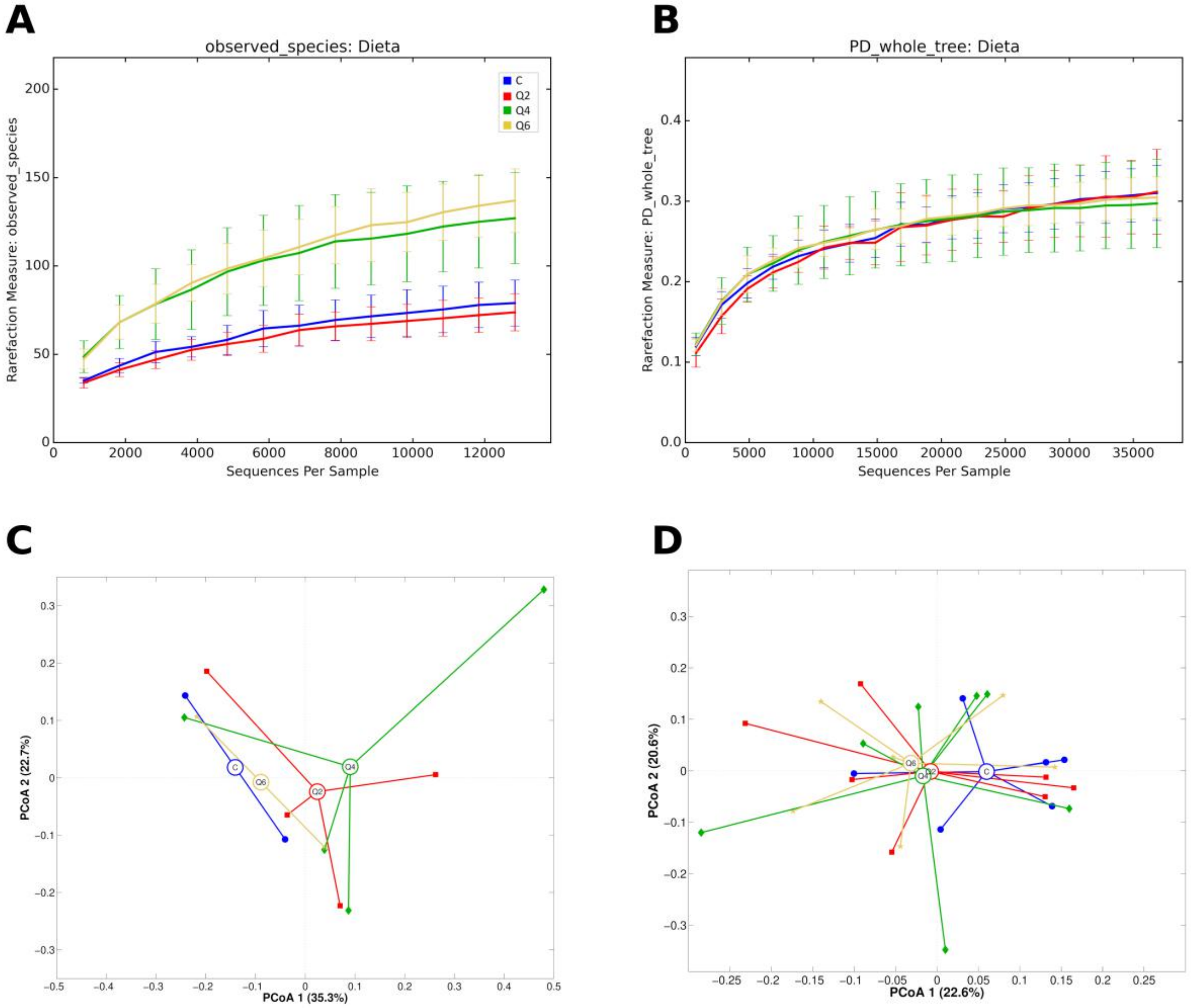

SUPPLEMENTARY TABLES

Supplementary Table S1. Table reporting the statistics of the 16S amplicon-based sequencing

Community	N samples	Total reads	Average reads	Average zOTU[1]	Range
Bacteria	26	3,490,275	134,241 ± 29,286	58,058 ± 16,316	21,927 – 89,537
Fungi	12	1,236,502	103,042 ± 31,419	62,214 ± 28,838	34,297 – 128,135
Protozoa	26	4,878,896	187,650 ± 60,619	117,827 ± 47,356	36,480 – 223,482

[1] zero-radius operational taxonomic unit

Supplementary Table S2. Relative abundance (%) of rumen bacterial and archaeal phyla. Taxa with at least one significant (*P*<0.05) effect are highlighted in red. "*" indicates statistically significant p-values.

	Diet[2]					*P*-value[4]		
**Phylum[1]**	**C**	**Q2**	**Q4**	**Q6**	**SEM[3]**	**T**	**Lin**	**Quadr**
Bacteria								
Firmicutes	34.8[a]	27.4[b]	28.5[ab]	34.5[a]	0.078	0.048*	0.963	0.008*
Bacteroidota	32.6	42.2	41.7	41.0	0.099	0.081	0.064	0.068
Verrucomicrobiota	6.1	7.4	7.4	5.3	0.156	0.349	0.555	0.096
Proteobacteria	4.3	5.2	2.9	4.1	0.331	0.563	0.621	0.780
Patescibacteria	3.5	4.4	4.2	3.5	0.336	0.872	0.998	0.427
Chloroflexi	1.7[a]	1.1[a]	1.2[a]	0.4[b]	0.353	0.007*	0.002*	0.115
Planctomycetota	0.9	0.4	0.5	0.3	0.311	0.102	0.027*	0.971
Synergistota	0.8[a]	0.3[a]	0.2[ab]	<0.1[b]	0.622	0.035*	0.005*	0.691
Fibrobacterota	0.5	0.9	0.6	0.6	0.375	0.600	0.679	0.400
Cyanobacteria	0.4	0.3	0.2	0.2	0.336	0.280	0.075	0.998
Firmicutes/ Bacteroidota ratio	1.14[a]	0.676[b]	0.771[b]	0.901[ab]	0.145	0.034*	0.208	0.011*
Archaea								
Euryarchaeota	5.7	3.5	4.5	2.5	0.232	0.062	0.025*	0.764
Thermoplasmatota	0.3[b]	0.7[a]	0.5[ab]	0.4[b]	0.281	0.026*	0.577	0.012*

[a-b]Mean values in the same row with different superscripts differ (P < 0.05) for the treatment effect.
[1]Data were log-transformed before statistics. The SEM associated with log-transformed data are in log scale. Only phyla with relative abundance greater than 0.5% before LS-means analysis in at least one treatment are reported.
[2]Diets with different levels of quebracho condensed tannin (CT) extract: C = no CT extract; Q2 = 2% CT extract on DM basis; Q4 = 4% CT extract; Q6 = 6% CT extract.
[3]Largest SEM.
[4]*P*-value of: T = treatment; Lin = linear effect of CT inclusion; Quadr = quadratic effect of CT inclusion.

Supplementary Table S3. Relative abundance (%) of rumen bacterial and archaeal classes. Taxa with at least one significant (*P*<0.05) effect are highlighted in red. "*" indicates statistically significant p-values.

	**Diet**[2]					***P*-value**[4]		
**Class**[1]	**C**	**Q2**	**Q4**	**Q6**	**SEM**[3]	**T**	**Lin**	**Quadr**
Bacteria								
Bacteroidia	32.6	42.2	41.7	41.0	0.099	0.081	0.064	0.068
Clostridia	26.3	20.0	19.8	23.9	0.108	0.091	0.473	0.018*
Negativicutes	6.5	5.8	7.2	8.5	0.121	0.109	0.056	0.183
Kiritimatiellae	5.9	7.0	7.1	5.0	0.161	0.386	0.509	0.119
Gammaproteobacteria	4.3	5.0	2.7	3.9	0.343	0.537	0.546	0.755
Saccharimonadia	3.0	4.0	4.0	3.6	0.349	0.861	0.692	0.468
Anaerolineae	1.7[a]	1.1[a]	1.2[a]	0.4[b]	0.353	0.007*	0.002*	0.115
Bacilli	1.0	1.29	1.1	1.7	0.157	0.077	0.029*	0.387
Planctomycetes	0.9	0.5	0.5	0.3	0.311	0.102	0.027*	0.971
Synergistia	0.8[a]	0.3[a]	0.2[ab]	<0.1[b]	0.622	0.035*	0.005*	0.691
Fibrobacteria	0.5	0.9	0.6	0.6	0.375	0.600	0.679	0.400
Lentisphaeria	0.1[b]	0.3[a]	0.3[a]	0.2[ab]	0.315	0.048*	0.347	0.012*
Archaea								
Methanobacteria	5.7	3.5	4.5	2.5	0.232	0.062	0.025*	0.764
Thermoplasmata	0.3[b]	0.7[a]	0.5[ab]	0.4[b]	0.281	0.026*	0.577	0.012*

[a-b]Mean values in the same row with different superscripts differ (P < 0.05) for the treatment effect.
[1]Data were log-transformed before statistics. The SEM associated with log-transformed data are in log scale. Only classes with relative abundance greater than 0.5% before LS-means analysis in at least one treatment are reported.
[2]Diets with different levels of quebracho condensed tannin (CT) extract: C = no CT extract; Q2 = 2% CT extract on DM basis; Q4 = 4% CT extract; Q6 = 6% CT extract.
[3]Largest SEM.
[4]*P*-value of: T = treatment; Lin = linear effect of CT inclusion; Quadr = quadratic effect of CT inclusion.

Supplementary Table S4. Relative abundance (%) of rumen bacterial and archaeal orders. Taxa with at least one significant (*P*<0.05) effect are highlighted in red. "*" indicates statistically significant p-values.

	Diet[2]					*P*-value[4]		
**Order[1]**	**C**	**Q2**	**Q4**	**Q6**	**SEM[3]**	**T**	**Lin**	**Quadr**
Bacteria								
Bacteroidales	32.5	42.0	41.6	40.9	0.099	0.080	0.061	0.069
Oscillospirales	13.7	9.8	9.4	11.2	0.121	0.082	0.187	0.025*
Lachnospirales	7.2	5.5	5.8	7.5	0.103	0.052	0.621	0.009*
WCHB1-41	5.9	7.0	7.1	5.0	0.161	0.386	0.509	0.119
Enterobacterales	4.2	4.9	2.2	3.3	0.384	0.450	0.392	0.745
Veillonellales-Selenomonadales	3.7	3.2	3.9	3.3	0.207	0.745	0.878	0.928
Saccharimonadales	3.0	4.0	4.0	3.7	0.349	0.861	0.692	0.468
Clostridia UCG-014	2.5	2.3	2.3	3.1	0.198	0.429	0.402	0.168
Acidaminococcales	2.2[b]	2.2[b]	2.7[b]	5.1[a]	0.182	0.016*	0.005*	0.073
Anaerolineales	1.7[a]	1.1[a]	1.2[a]	0.4[b]	0.353	0.007*	0.002*	0.115
Christensenellales	1.7	1.1	1.3	1.0	0.172	0.188	0.096	0.595
Pirellulales	0.9	0.5	0.5	0.3	0.311	0.971	0.027*	0.971
Synergistales	0.8[a]	0.3[a]	0.2[ab]	<0.1[b]	0.622	0.035*	0.005*	0.691
Clostridia (other)	0.6	0.4	0.4	0.3	0.264	0.438	0.140	0.630
Fibrobacterales	0.5	0.9	0.6	0.6	0.375	0.600	0.679	0.400
Erysipelotrichales	0.5	0.5	0.4	1.0	0.153	0.009*	0.026*	0.010*
Archaea								
Methanobacteriales	5.7	3.5	4.5	2.5	0.232	0.062	0.025*	0.764
Methanomassiliicoccales	0.3[b]	0.7[a]	0.5[ab]	0.4[b]	0.028	0.026*	0.577	0.012*

[a-b]Mean values in the same row with different superscripts differ (P < 0.05) for the treatment effect.
*$p < 0.05$.
[1]Data were log-transformed before statistics. The SEM associated with log-transformed data are in log scale. Only orders with relative abundance greater than 0.5% before LS-means analysis in at least one treatment are reported.
[2]Diets with different levels of quebracho condensed tannin (CT) extract: C = no CT extract; Q2 = 2% CT extract on DM basis; Q4 = 4% CT extract; Q6 = 6% CT extract.
[3]Largest SEM.
[4]*P*-value of: T = treatment; Lin = linear effect of CT inclusion; Quadr = quadratic effect of CT inclusion.

Supplementary Table S5. Relative abundance (%) of rumen bacterial and archaeal families.Taxa with at least one significant (*P*<0.05) effect are highlighted in red. "*" indicates statistically significant p-values.

	Diet[2]					*P*-value[4]		
**Family[1]**	**C**	**Q2**	**Q4**	**Q6**	**SEM[3]**	**T**	**Lin**	**Quadr**
Bacteria								
Prevotellaceae	20.7	27.3	26.6	27.7	0.101	0.375	0.045*	0.287
Lachnospiraceae	7.07	5.4	5.8	7.4	0.102	0.053	0.604	0.053
Kirimatellae (WCHB1-41 group)	4.6	5.4	5.1	3.9	0.240	0.783	0.599	0.388
Oscillospiraceae	4.6	3.5	3.9	4.4	0.139	0.464	0.963	0.146
Succinivibrionacea	3.7	4.7	2.1	3.1	0.392	0.484	0.463	0.888
Selenomonadaceae	3.6	3.2	3.9	3.3	0.206	0.745	0.889	0.916
Ruminococcaceae	3.4	2.2	1.9	2.1	0.186	0.162	0.085	0.982
Rikenellaceae	3.1	4.6	4.4	4.0	0.180	0.311	0.339	0.125
Bacteroidales (F082 group)	3.0	2.0	2.5	1.9	0.202	0.394	0.674	0.394
Bacteroidales (RF16 group)	3.0	6.3	4.3	6.1	0.271	0.143	0.130	0.374
Saccharimonadaceae	3.0	4.0	4.0	3.6	0.349	0.861	0.692	0.468
Acidaminococcaceae	2.2[b]	2.2[b]	2.7[b]	5.1[a]	0.182	0.016*	0.005*	0.070
Clostridia (UCG-014 group)	2.0	1.8	1.8	2.3	0.202	0.541	0.532	0.205
Oscillospirales (UCG-011 group)	1.8[a]	1.2[a]	1.0[a]	0.3[b]	0.341	0.016*	0.003*	0.410
Anaerolineaceae	1.7[a]	1.1[a]	1.2[a]	0.4[b]	0.353	0.007*	0.002*	0.115
Christensenellacea	1.7	1.1	1.3	1.0	0.172	0.188	0.096	0.595
Eubacterium coprostanoligenes group	1.4	1.2	1.3	1.9	0.258	0.458	0.344	0.473
Muribaculaceae	1.1[a]	0.5[a]	0.9[a]	0.1[b]	0.452	0.008*	0.003*	0.050
Hungateiclostridiaceae	0.9	0.9	0.7	1.2	0.200	0.087	0.446	0.046*
Pirellulaceae	0.9	0.5	0.5	0.3	0.311	0.102	0.027*	0.971
Synergistaceae	0.8[a]	0.3[a]	0.2[ab]	<0.1[b]	0.622	0.035*	0.005*	0.691
WCHB1-41 (other)	0.8	0.8	1.0	0.6	0.133	0.071	0.368	0.049*
Clostridia (other)	0.6	0.4	0.4	0.3	0.264	0.438	0.140	0.630
Fibrobacteraceae	0.5	0.9	0.6	0.6	0.375	0.600	0.679	0.400
Erysipelotrichaceae	0.5[ab]	0.4[b]	0.3[b]	0.8[a]	0.178	0.018*	0.160	0.005*
Unclassified WCHB1-41	0.3	0.3	0.4	0.23	0.301	0.636	0.668	0.516
Archaea								
Methanobacteriaceae	5.7	3.5	4.5	2.5	0.232	0.062	0.025*	0.764
Methanomethylophilaceae	0.3[b]	0.7[a]	0.5[ab]	0.4[b]	0.281	0.026*	0.577	0.012*

[a-b]Mean values in the same row with different superscripts differ (P < 0.05) for the treatment effect.
[1]Data were log-transformed before statistics. The SEM associated with log-transformed data are in log scale. Only families with relative abundance greater than 0.5% before LS-means analysis in at least one treatment are reported.
[2]Diets with different levels of quebracho condensed tannin (CT) extract: C = no CT extract; Q2 = 2% CT extract on DM basis; Q4 = 4% CT extract;
Q6 = 6% CT extract. [3]Largest SEM. [4]*P*-value of: T = treatment; Lin = linear effect of CT inclusion; Quadr = quadratic effect of CT inclusion.

Supplementary Table S6. Relative abundance (%) of rumen bacterial and archaeal genera. Taxa with at least one significant (*P*<0.05) effect are highlighted in red. "*" indicates statistically significant p-values.

	Diet[2]					*P*-value[4]		
**Genus[1]**	**C**	**Q2**	**Q4**	**Q6**	**SEM[3]**	**T**	**Lin**	**Quadr**
Bacteria								
Prevotella	15.7	20.8	19.9	17.7	0.111	0.121	0.424	0.031*
Uncultured rumen bacterium (WCHB1-41)	4.6	5.4	5.1	3.9	0.240	0.783	0.599	0.388
NK4A214 group	4.0	3.0	3.2	3.7	0.172	0.624	0.874	0.215
Candidatus Saccharimonas	3.0	4.0	4.0	3.6	0.349	0.861	0.692	0.468
Rikenellaceae RC9 gut group	3.0	4.6	4.4	3.9	0.179	0.271	0.329	0.103
Succiniclasticum	2.2^{b}	2.20	2.7^{b}	5.1^{a}	0.182	0.016*	0.005*	0.073
Clostridia UCG-014 (other)	2.0	1.8	1.8	2.3	0.202	0.541	0.532	0.205
Bacteroidales RF16 group (other)	1.9	3.5	2.5	2.3	0.319	0.355	0.823	0.176
Succinivibrionaceae UCG-002	1.9	2.7	1.2	1.1	0.518	0.528	0.303	0.669
Uncultured rumen bacterium (UCG-011)	1.8^{a}	1.2^{a}	1.0^{a}	0.3^{b}	0.341	0.016*	0.003*	0.259
Flexilinea	1.7^{a}	1.1^{a}	1.2^{a}	0.4^{b}	0.353	0.007*	0.002*	0.115
F082 (other)	1.7	0.8	1.0	0.9	0.269	0.205	0.196	0.221
Christensenellaceae R-7 group	1.6	1.1	1.3	1.0	0.181	0.248	0.109	0.686
Ruminococcaceae (other)	1.5	0.6	0.6	0.4	0.395	0.149	0.039*	0.405
Ruminococcus	1.4	1.2	1.0	1.1	0.190	0.691	0.365	0.574
Prevotellaceae UCG-001	1.2^{b}	2.1^{ab}	2.5^{a}	3.6^{a}	0.195	0.015*	0.002*	0.634
Prevotellaceae UCG-003	1.2	1.4	1.5	1.4	0.150	0.776	0.503	0.472
Prevotellaceae (other)	1.2	1.6	1.3	1.8	0.169	0.354	0.220	0.977
Quinella	1.1	1.4	1.4	0.6	0.548	0.383	0.298	0.180
Uncultured rumen bacterium (Muribaculaceae)	1.0^{a}	0.5^{a}	0.7^{a}	0.1^{b}	0.540	0.013*	0.004*	0.088
p-1088-a5 gut group	0.9	0.5	0.5	0.3	0.314	0.125	0.034*	0.982
Saccharofermentans	0.9	0.9	0.7	1.0	0.210	0.066	0.342	0.046*
Lachnospiraceae (other)	0.8	0.7	0.9	1.0	0.012	0.215	0.161	0.300
WCHB1-41 (other)	0.8	0.8	1.0	0.6	0.133	0.071	0.368	0.049*
[Eubacterium] coprostanoligenes group (other)	0.7^{ab}	0.6^{bc}	0.4^{c}	0.9^{a}	0.191	0.011*	0.390	0.004*
Lachnospiraceae AC2044 group	0.7	0.8	0.5	0.9	0.248	0.264	0.840	0.332
Lachnospiraceae XPB1014 group	0.7^{a}	0.4^{a}	0.4^{a}	0.1^{b}	0.349	0.021*	0.004*	0.337
Selenomonadaceae (other)	0.7	0.6	0.7	1.0	0.263	0.196	0.160	0.134
Unclassified F082	0.7	0.5	0.6	0.3	0.289	0.121	0.056	0.278
Veillonellaceae UCG-001	0.7	0.6	0.7	0.7	0.141	0.439	0.341	0.460
Acetitomaculum	0.6	0.3	0.4	0.5	0.184	0.190	0.604	0.068
Clostridia (other)	0.6	0.4	0.4	0.3	0.264	0.438	0.140	0.630
[Eubacterium] ruminantium group	0.6	0.5	0.6	0.7	0.188	0.296	0.414	0.290
Fretibacterium	0.6	0.4	0.7	<0.01	0.941	0.080	0.020*	0.112

Lachnospiraceae NK3A20 group	0.6	0.3	0.4	0.6	0.220	0.160	0.765	0.043*
Ruminobacter	0.6	0.9	0.3	1.3	0.775	0.550	0.669	0.502
Unclassified Bacteroidales RF16 group	0.6^{b}	1.2^{ab}	0.7^{b}	2.8^{a}	0.326	0.019*	0.011*	0.348
Uncultured rumen bacterium (F082)	0.6	0.5	0.6	0.5	0.180	0.513	0.437	0.845
Butyrivibrio	0.5^{b}	0.4^{b}	0.4^{b}	0.9^{a}	0.179	0.006*	0.042*	0.002*
Fibrobacter	0.5	0.9	0.6	0.6	0.375	0.600	0.679	0.400
Pseudobutyrivibrio	0.5	0.5	0.5	0.5	0.197	0.983	0.806	0.771
Uncultured rumen bacterium (Bacteroidales RF16 group)	0.5^{b}	1.3^{a}	0.9^{ab}	0.5^{b}	0.286	0.028*	0.809	0.005*
Unclassified [Eubacterium] coprostanoligenes group	0.4	0.3	0.4	0.4	0.455	0.936	0.877	0.893
Unclassified WCHB1-41	0.3	0.3	0.4	0.2	0.301	0.636	0.668	0.516
Prevotella_9	0.2	0.3	0.4	0.8	0.437	0.132	0.022*	0.818
Prevotellaceae UCG-004	0.2	0.2	0.2	0.6	0.286	0.056	0.014*	0.261
UCG-002	0.2	0.2	0.3	0.4	0.349	0.529	0.155	0.824
Archaea								
Methanobrevibacter	5.5	3.4	4.3	2.3	0.231	0.054	0.021*	0.716
Uncultured (Methanomethylophilaceae)	0.3^{b}	0.7^{a}	0.4^{b}	0.4^{b}	0.278	0.013*	0.770	0.007*

[a-b]Mean values in the same row with different superscripts differ ($P < 0.05$) for the treatment effect.
[1]Data were log-transformed before statistics. The SEM associated with log-transformed data are in log scale. Only genera with relative abundance greater than 0.5% before LS-means analysis in at least one treatment are reported.
[2]Diets with different levels of quebracho condensed tannin (CT) extract: C = no CT extract; Q2 = 2% CT extract on DM basis; Q4 = 4% CT extract; Q6 = 6% CT extract.
[3]Largest SEM.
[4]*P*-value of: T = treatment; Lin = linear effect of CT inclusion; Quadr = quadratic effect of CT inclusion.

Supplementary Table S7. Relative abundances of the fungal genera in the rumen microbiota. The mean abundance values for each taxon are reported in the middle column for each diet (C, Q2, Q4, Q6).

**Taxon**	**C**	**Q2**	**Q4**	**Q6**
Neocallimastix	54.2	55.3	44.3	34.5
Caecomyces	14.8	16.6	12.6	10.6
Feramyces	10.1	9.8	13.9	41.0
Neocallimastigaceae (other)	8.9	5.5	4.9	5.1
Piromyces	5.9	6.7	4.8	3.6
Liebetanzomyces	5.2	2.3	1.6	1.1
Fungi (other)	0.3	0.4	1.3	1.3
Orpinomyces	0.0	2.9	14.1	0.0
Aspergillus	0.0	0.0	0.3	0.8

Supplementary Table S8. Relative abundance (%) of rumen protozoal genera. Taxa with at least one significant (*P*<0.05) effect are highlighted in red. "*" indicates statistically significant p-values.

	Diet[2]					*P*-value		
**Genus[1]**	**C**	**Q2**	**Q4**	**Q6**	**SEM[3]**	**T**	**Lin**	**Quadr**
Entodinium	47.1	54.7	43.5	52.0	0.115	0.244	0.871	0.871
Trichostomatia (other)	37.4	26.6	32.6	25.3	0.177	0.363	0.230	0.781
Ciliophora (other)	7.4	5.5	10.0	11.4	0.270	0.224	0.138	0.359
Diplodinium	1.7[a]	0.9[ab]	0.5[b]	0.2[c]	0.409	0.006*	<0.001*	0.486
Ophryoscolex	0.6	0.5	1.0	0.74	0.086	0.223	0.268	0.838
Isotricha	0.4[ab]	0.2[b]	0.4[b]	1.3[a]	0.370	0.026*	0.038*	0.016*
Polyplastron	0.5	0.6	1.0	1.1	0.287	0.151	0.037*	0.931
Unclassified Trichostomatia	0.4	0.1	0.2	0.2	0.624	0.466	0.750	0.230
Eukaryota (other)	0.1	0.1	0.5	0.6	0.716	0.071	0.015*	0.584

[a-b]Mean values in the same row with different superscripts differ (P < 0.05) for the treatment effect.
[1]Data were log-transformed before statistics. The SEM associated with log-transformed data are in log scale. Only genus with relative abundance greater than 0.5% before LN-means analysis in at least one treatment are reported.
[2]Diets with different levels of quebracho condensed tannin (CT) extract: C = no CT extract; Q2 = 2% CT extract on DM basis; Q4 = 4% CT extract; Q6 = 6% CT extract.
[3]Largest SEM.
[4]*P*-value of: T = treatment; Lin = linear effect of CT inclusion; Quadr = quadratic effect of CT inclusion.
